# LIMK1 promotes peritoneal metastasis of gastric cancer and is a therapeutic target

**DOI:** 10.1038/s41388-021-01656-1

**Published:** 2021-04-21

**Authors:** Xi Kang, Weilin Li, Weixin Liu, Han Liang, Jingyu Deng, Chi Chun Wong, Sinan Zhao, Wei Kang, Ka Fai To, Philip Wai Yan Chiu, Guiying Wang, Jun Yu, Enders Kwok Wai Ng

**Affiliations:** 1 Institute of Digestive Disease and Department of Medicine and Therapeutics, State Key Laboratory of Digestive Disease, Li Ka Shing Institute of Health Sciences, The Chinese University of Hong Kong, Hong Kong, China; 2 Department of Surgery, Hebei Medical University 4th Hospital, Shijiazhuang, China; 3 Department of Surgery, The Chinese University of Hong Kong, Hong Kong, China; 4 Department of Gastroenterology, Tianjin Medical University Cancer Hospital, City Key Laboratory of Tianjin Cancer Center and National Clinical Research Center for Cancer, Tianjin, China; 5 Department of Endocrinology, Hebei Medical University 2nd Hospital, Shijiazhuang, China; 6 Department of Anatomical and Cellular Pathology, The Chinese University of Hong Kong, Hong Kong, China

**Keywords:** Metastasis, Molecular biology

## Abstract

Peritoneal metastasis is a common form of metastasis among advanced gastric cancer patients. In this study, we reported the identification of LIM domain kinase 1 (LIMK1) as a promoter of gastric cancer peritoneal metastasis, and its potential to be a therapeutic target of dabrafenib (DAB). Using transcriptomic sequencing of paired gastric cancer peritoneal metastasis, primary tumors, and normal gastric tissues, we first unveiled that LIMK1 is selectively up-regulated in metastatic tumors. Increased LIMK1 in gastric cancer peritoneal metastasis was validated by immunohistochemistry analysis of an independent patient cohort. In vitro functional studies demonstrated that LIMK1 knockout or knockdown significantly inhibited cell migration and invasion of gastric cancer cells. LIMK1 knockout also abrogated peritoneal and liver metastases of gastric cancer cells in nude mice in vivo. Dabrafenib, a small molecule targeting LIMK1, was found to decrease cell migration and invasion of gastric cancer cells in vitro and abolish peritoneal and liver metastasis formation in vivo. Mechanistically, either LIMK1 knockout or Dabrafenib inhibited LIMK1 expression and phosphorylation of its downstream target cofilin. Taken together, our results demonstrated that LIMK1 functions as a metastasis promoter in gastric cancer by inhibiting LIMK1-p-cofilin and that Dabrafenib has the potential to serve as a novel treatment for gastric cancer peritoneal metastasis.

## Introduction

Gastric cancer remains one of the most common cancer and the third leading cause of cancer-related deaths worldwide, with over a million new cases and an estimated 783,000 deaths each year [1]. Gastric cancer is asymptomatic in the early stages and about 80–90% of gastric cancer patients are diagnosed at advanced stage with metastasis [2]. Peritoneal metastasis is one of the most common form of metastasis in gastric cancer. It is found in up to 14% of newly diagnosed gastric cancer patients, and is also the most common site (~50%) of recurrence in gastric cancer patients after radical surgery [3, 4]. Due to the lack of effective treatment, the median survival of patients with peritoneal metastasis was only 3–6 months [3, 5]. As a consequence, the prognosis of gastric cancer remains poor. However, the molecular mechanisms underlying the occurrence of peritoneal metastasis of gastric cancer remains poorly understood. Hence, investigation of the molecular mechanism underlying gastric cancer peritoneal metastasis is warranted.

Molecular features of cancer metastasis are distinct from that of primary tumor. Accumulating evidence indicates that tumor metastases harbor unique gene expression profiles compared to primary tumors in multiple cancer types, such as breast cancer, uterine sarcoma, and renal carcinomas [6–10]. The molecular profiling of tumor metastasis is thus important for the discovery of metastasis-specific drug targets and prognostic biomarkers. However, there has been very limited research on the molecular characterization of gastric cancer peritoneal metastasis. To unveil molecular basis of gastric cancer peritoneal metastasis, we performed the transcriptomic sequencing of paired peritoneal metastases, primary tumors, and normal stomach tissues from gastric cancer patients.

Analysis of our sequencing dataset revealed for the first time that LIM domain kinase 1 (LIMK1) is selectively up-regulated in peritoneal metastases compared to primary tumors and normal stomach tissues. LIMK1 is a serine/threonine-protein kinase that plays an essential role in the regulation of actin filament dynamics [11, 12], however; its role in gastric cancer peritoneal metastasis is largely unknown. We therefore determined the role of LIMK1 in gastric cancer peritoneal metastasis. We demonstrated that LIMK1 promotes gastric cancer cell migration and invasion, leading to enhanced peritoneal metastasis in mice models. We also first showed that Dabrafenib, an inhibitor of LIMK1, repressed gastric cancer cell migration and invasion in vitro and peritoneal metastasis in vivo. These data support a functional role of LIMK1 in gastric cancer peritoneal metastasis and suggest a novel therapeutic approach for its treatment.

## Results

### Transcriptomic sequencing identified unique gene expression profiles of gastric cancer peritoneal metastasis

**Fig. 1:**
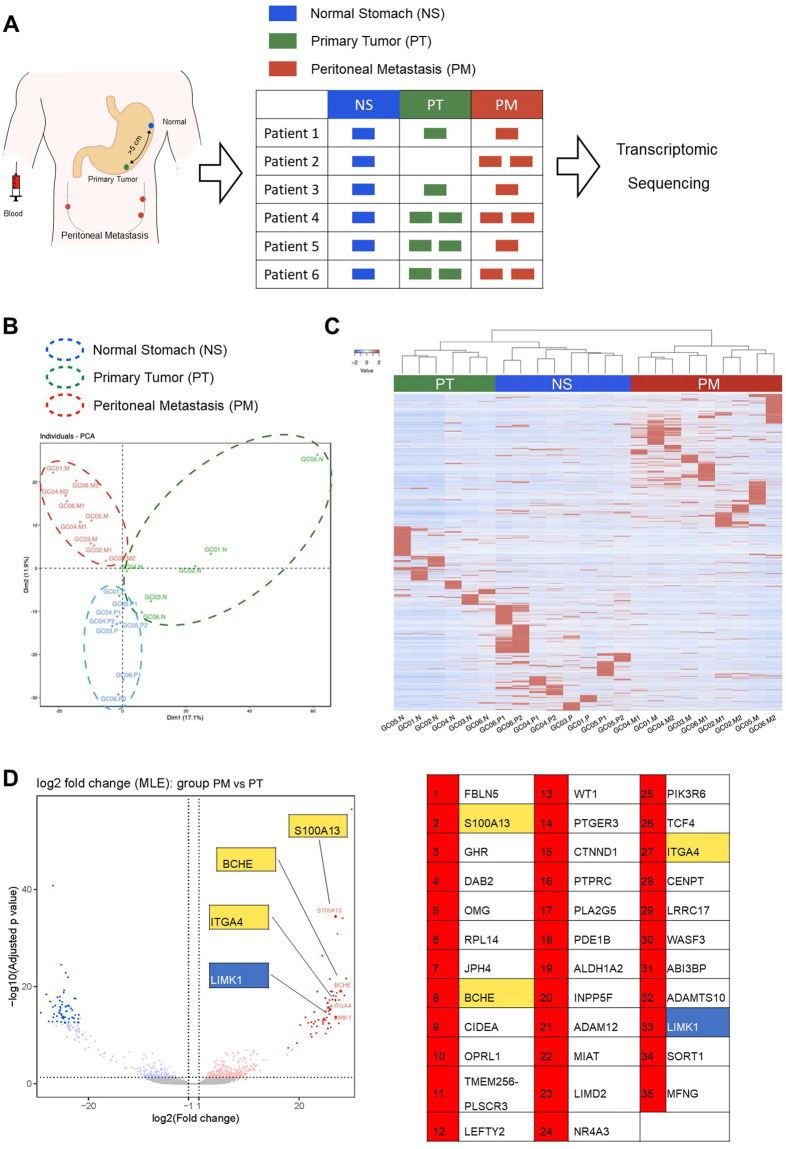
Transcriptomic sequencing identified unique gene expression profiles of gastric cancer peritoneal metastasis compared to primary gastric cancer and normal stomach tissue. **A** Paired normal stomach (NS), primary tumors (PT), and peritoneal metastases (PM) were collected from 6 gastric cancer patients and submitted for transcriptome sequencing. **B** PCA and (**C**) unsupervised, hierarchical clustering of normalized counts from transcriptome sequencing of NS, PT, and PM. **D** Four candidate genes (S100A13, BCHE, ITGA4, and LIMK1) were selected from top up-regulated genes in peritoneal metastases compared to primary gastric cancer. All four genes have been reported having oncogenic functions and have inhibitor(s) available.

We have collected paired normal stomach tissues, primary gastric tumors, and peritoneal metastasis from 6 gastric cancer patients (Fig. 1A) and performed transcriptomic sequencing using Illumina HiSeq (Fig. 1A). PCA analysis and non-supervised clustering of the transcriptome datasets showed that peritoneal metastases have distinct gene expression profiles compared to primary gastric tumors or normal stomach tissues (Fig. 1B and 1C).

Next, we aim to identify genes uniquely up-regulated in peritoneal metastasis that could be targeted by readily available drugs. According to these criteria, 4 candidate genes were selected from the top up-regulated genes in peritoneal metastasis compared to primary gastric cancer, which were S100A13 (S100 calcium-binding protein A13), BCHE (Butyrylcholinesterase), ITGA4 (Integrin alpha 4) and LIMK1 (LIM domain kinase 1) (Fig. 1D). All these genes are reported to have oncogenic function and have commercially available inhibitors [13–16]. Collectively, our data suggested that the gene expression profile is markedly different in peritoneal metastases compared to primary gastric tumors and normal stomach tissue, implying that peritoneal metastases have evolved differential mechanisms to promote growth and survival that could potentially be targeted for its treatment.

### LIMK1 is overexpressed in peritoneal metastases compared to primary tumors in gastric cancer

**Fig. 2:**
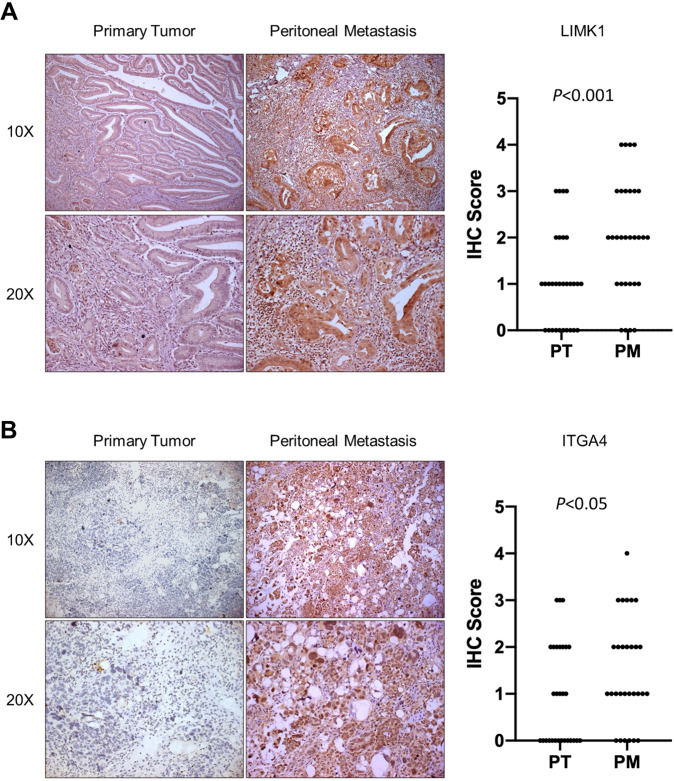
IHC staining of samples from validation cohort showed that LIMK1 and ITGA4 were overexpressed in peritoneal metastases (PM) compared to primary tumors (PT). **A** LIMK1 was significantly up-regulated in PM compared to PT (*P* = 0.0002, *n* = 29). **B** ITGA4 was also significantly up-regulated in PM compared to PT (*P* = 0.0239, *n* = 29).

To validate the differential gene expression of the selected candidates between peritoneal metastases and primary gastric tumors from gastric cancer patients, we have collected an independent cohort of gastric cancer patients (*N* = 29) with paired peritoneal metastases and primary tumors. We examined the expression of S100A13, BCHE, ITGA4, and LIMK1 using immunohistochemistry staining (Fig. 2). Among the 4 candidates, LIMK1 (Fig. 2A, *P* < 0.001) and ITGA4 (Fig. 2B, *P* < 0.05) expression were increased in the peritoneal metastases compared to primary tumors, while expression of S100A13 and BCHE (Fig. S1) were not significantly different between peritoneal metastases and primary tumors. These results suggested that LIMK1 and ITGA4 are overexpressed in peritoneal metastases compared to primary tumors, suggesting the potential correlation with peritoneal metastasis in gastric cancer. To determine the cause of LIMK1 in GC, we analyzed LIMK1 promoter methylation and copy number variations in TCGA GC cohort (Fig. S2). Promoter methylation showed no significant correlation with LIMK1 mRNA; but we observed a positive correlation between LIMK1 and copy number gain (Fig. S2), implying that copy number gain plays a role in mediating LIMK1 overexpression in GC.

### LIMK1 knockout or knockdown suppresses gastric cancer cell migration and invasion

**Fig. 3:**
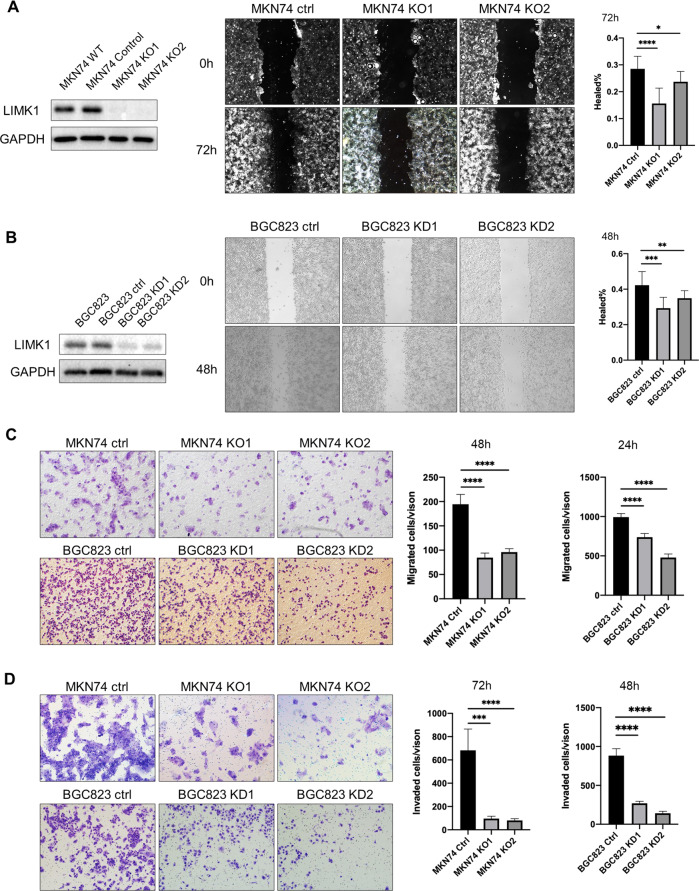
LIMK1 knockout/knockdown significantly reduced cell migration and invasion of gastric cancer cells. **A** LIMK1 knockout in MKN74 cells delayed wound closure (KO1 *P* < 0.0001, KO2 *P* < 0.05) after 72 h. **B** Knockdown of LIMK1 in BGC823 cells delayed wound closure (KD1 *P* = 0.0001, KD2 *P* < 0.01) after 48 h. **C** LIMK1 knockout or knockdown in MKN74 (KO1 *P* < 0.0001, KO2 *P* < 0.0001) or BGC823 (KD1 *P* < 0.0001, KD2 *P* < 0.0001) cells, respectively, decreased cell migration in transwell migration assay after 48 and 24 h, respectively. **D** LIMK1 knockout or knockdown in MKN74 (KO1 *P* < 0.001, KO2 *P* < 0.0001) or BGC823 (KD1 *P* < 0.0001, KD2 *P* < 0.0001) cells, respectively, decreased cell invasion in transwell invasion assay after 72 and 48 h, respectively.

Because of more significant overexpression of LIMK1 in peritoneal metastases, we selected this gene for further evaluation. To investigate biological function of LIMK1 in gastric cancer, we performed CRISPR/Cas9 to knockout LIMK1 in MKN74 cells with 2 sgRNA. Knockout efficiency was validated by Western blot (Fig. 3A). In addition, we used 2 siRNAs to knockdown LIMK1 expression in BGC823 cells, and knockdown efficiency was also validated (Fig. 3B).

We next performed wound healing and transwell migration assays to assess the effect of LIMK1 on gastric cancer cell migration. A delay in wound closure was observed in both MKN74 and BGC823 cells after knockout or knockdown of LIMK1 (Fig. 3A and 3B). In transwell migration assay, both MKN74 and BGC823 cells showed a decrease in the number of migrated cells after LIMK1 knockout or knockdown (Fig. 3C). Transwell matrigel invasion assay was next performed to assess the effect of LIMK1 on gastric cancer cell invasion. Knockout or knockdown of LIMK1 in MKN74 and BGC823 cells, respectively, significantly decreased cell invasion through the invasion chamber (Fig. 3D). These results indicated that LIMK1 is functionally important for gastric cancer cell migration and invasion in vitro.

### Dabrafenib suppresses gastric cancer cell migration and invasion

In light of the role of LIMK1 in promoting cell migration and invasion in gastric cancer cells, we further investigated whether LIMK1 inhibitors could suppress cell migration and invasion in vitro. We chose 2 established inhibitors of LIMK1, Dabrafenib and diallyl disulfide (DADS). Dabrafenib an FDA-approved inhibitor of mutant BRAF(V600) metastatic melanoma, but it can also target LIMK1 with nanomolar potency [17]. DADS is an organosulfur compound found in garlic, which was reported to inhibit LIMK1 by downregulating Rac1-ROCK1/PAK1-LIMK1-ADF/cofilin pathway [18–20].

**Fig. 4:**
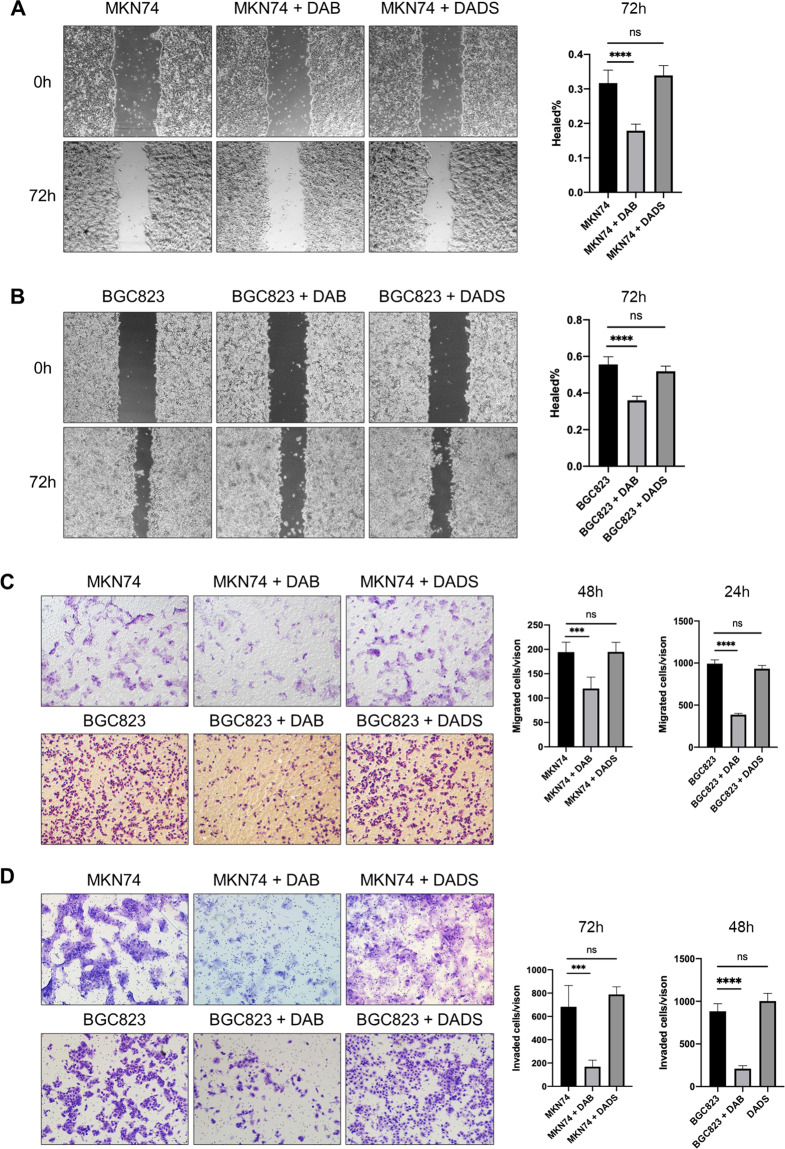
Dabrafenib (DAB), but not diallyl disulfide (DADS), suppressed cell migration and invasion of gastric cancer cells. **A** DAB (10 μM) treated MKN74 cells showed delayed wound closure (*P* < 0.0001) after 72 h. DADS (45 mg/L) did not impact wound closure. **B** DAB (10 μM) treated BGC823 cells showed delayed wound closure (*P* < 0.0001) after 72 h. DADS (45 mg/L) did not impact wound closure. **C** DAB (10 μM) treated MKN74 (*P* < 0.001) and BGC823 (*P* < 0.0001) cells showed decreased cell migration in transwell migration assay after 48 and 24 h, respectively. DADS (45 mg/L) had no effect on transwell migration. **D** DAB (10 μM) treated MKN74 (*P* < 0.001) and BGC823 (*P* < 0.0001) cells showed decreased cell invasion in transwell invasion assay after 72 and 48 h, respectively. DADS (45 mg/L) did not impact the transwell invasion of MKN74 or BGC823 cells.

We performed wound healing assay and transwell migration assay to evaluate the effect of Dabrafenib and DADS on gastric cancer cell migration. The wound closure of MKN74 and BCG823 cells treated with Dabrafenib was significantly reduced, but DADS had no significant effect (Fig. 4A and 4B). Consistent results were obtained in transwell migration assay, where only Dabrafenib could suppress migrated cell number significantly (Fig. 4C). We next determined cell invasion with transwell matrigel invasion assay. Dabrafenib, but not DADS, significantly decreased cell invasion of MKN74 and BGC823 cells (Fig. 4D). The results indicated that Dabrafenib, but not DADS, decreases cell migration and invasion ability of gastric cancer cells.

### LIMK1 knockout or Dabrafenib inhibit phosphorylation of the downstream target cofilin

**Fig. 5:**
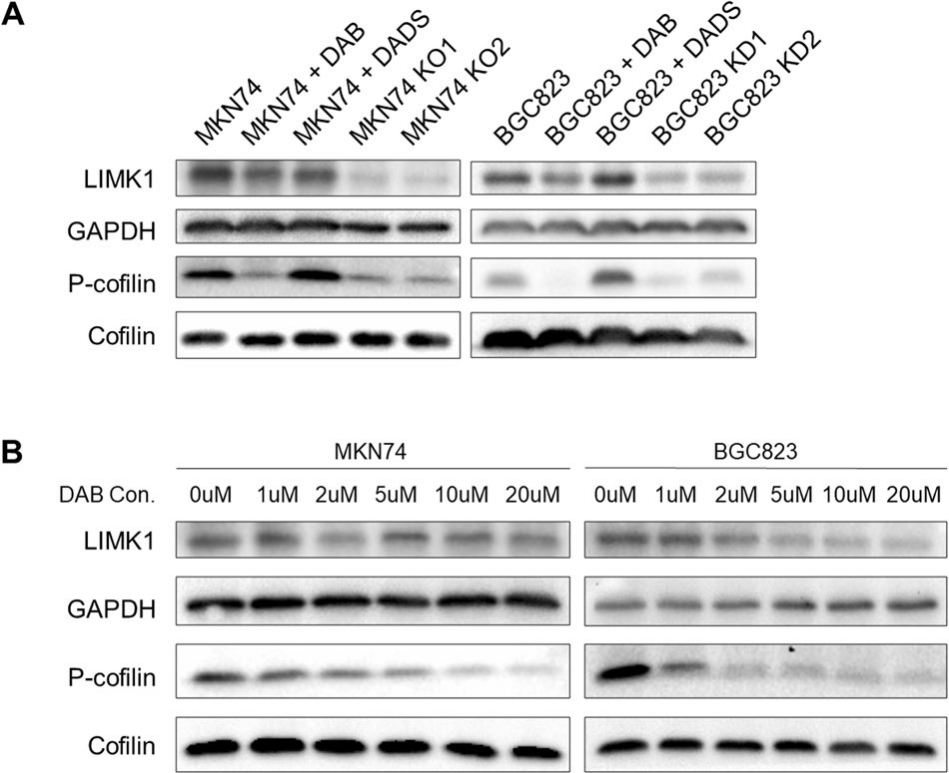
Dabrafenib (DAB) inhibits LIMK1 and reduces the phosphorylation of cofilin in a dose dependent manner. **A** DAB treatment for 48 h decreased p-cofilin level in MKN74 (left panel) and BGC823 (right panel) cells to an extent comparable to LIMK1 knockout or knockdown. **B** MKN74 (left panel) and BGC823 (right panel) cells were treated by different concentrations of DAB (0–20 μM). Levels of p-cofilin were dose-dependently decreased in both MKN74 and BGC823 cells.

LIMK1 phosphorylates and inactivates actin depolymerizing factors ADF/cofilin, resulting in stabilization of the actin cytoskeleton [11, 12]. We thus investigated whether LIMK1 knockout or pharmacological inhibition inhibit phosphorylation of cofilin. Knockout or knockdown of LIMK1 suppressed cofilin phosphorylation in MKN74 and BGC823 cells, respectively (Fig. 5A). Dabrafenib treatment also reduced LIMK1 expression and abolished p-cofilin expression in both cell lines (Fig. 5A). In line with its lack of effect on cell migration and invasion, DADS treatment failed to modulate LIMK1 or p-cofilin protein expression (Fig. 5A). We next treated MKN74 and BGC823 cells with different doses of Dabrafenib for 48 h. Western blot showed that p-cofilin levels were dose-dependently decreased by Dabrafenib (Fig. 5B). Our results imply that LIMK1 inhibition suppressed phosphorylation of cofilin, subsequently reducing cell migration and invasion ability of gastric cancer cells.

### LIMK1 knockout or Dabrafenib inhibits metastasis of gastric cancer cells in vivo

**Fig. 6:**
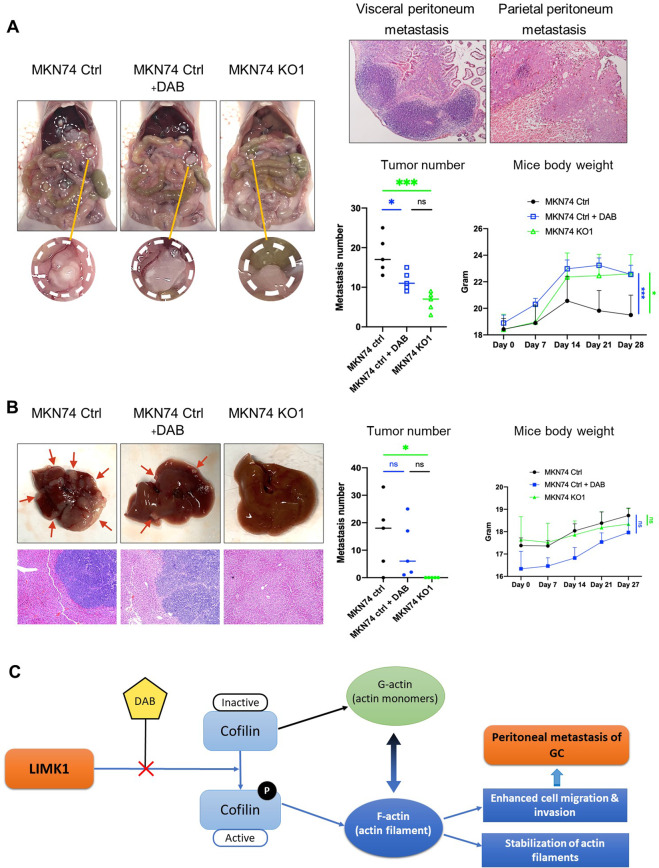
LIMK1 knockout or Dabrafenib (DAB) treatment reduced peritoneal and liver metastases in nude mouse xenograft models. **A** Representative pictures of peritoneal metastasis mouse model showing decreased metastases formation in DAB treated and LIMK1 knockout MKN74 cells (left panel). H&E staining of visceral and parietal peritoneum metastasis were shown on upper right panel. The number of peritoneal metastasis in LIMK1 knockout (*P* < 0.001) and DAB (*P* < 0.05) treated MKN74 cells was significantly reduced, and mice body weight of these groups were significantly higher than control group (lower right panel). **B** Representative pictures of liver metastasis nude mouse model showed decreased metastases formation in DAB treated and LIMK1 knockout MKN74 cells (upper left panel). H&E staining of liver with or without metastasis were shown (lower left panel). The number of liver metastases in MKN74 cells with LIMK1 knockout was significantly decreased (*P* < 0.05). The number of liver metastases in mice treated by DAB also showed a decreasing trend Average tumor number 15.6 (control) vs 10.2 (DAB treated) (right panel). **C** Molecular mechanism of LIMK1 and DAB in gastric cancer peritoneal metastasis. LIMK1 phosphorylates cofilin, subsequently increasing the level of F-actin, and leading to stabilization of actin filaments to promote gastric cancer cell migration and invasion. DAB inhibits LIMK1 and decreases p-cofilin, thereby suppressing the cell migration/invasion and metastasis capability of gastric cancer cells.

To validate the biological function of LIMK1 in gastric cancer metastasis in vivo, we performed experimental metastasis assays in nude mice. We first performed the peritoneal metastasis model by injecting MKN74 cells with or without LIMK1 knockout into abdominal cavity of nude mice. Mice injected with control MKN74 cells were given Dabrafenib or vehicle DMSO treatment by intraperitoneal (IP) injection every 3 days. After 28 days, metastasis in peritoneum was determined. As shown in Fig. 6A, LIMK1 knockout or Dabrafenib treatment both reduced the number of peritoneal metastases significantly compared to control. Body weight of mice in control group were also significantly lower than other 2 groups (Fig. 6A), indicating poor health. We next examined the role of LIMK1 in the liver metastasis model. MKN74 cells with or without LIMK1 knockout were given to nude mice via intrasplenic injection, and control MKN74 injected mice were also treated with vehicle or Dabrafenib (i.p., every 3 days). Liver metastases of LIMK1 knockout MKN74 cells was significantly suppressed compared to control MKN74 cells (Fig. 6B). Moreover, mice treated with Dabrafenib also showed a decreasing trend of liver metastases formation (Fig. 6B). Taken together, these results indicate that LIMK1 knockout or its pharmacological inhibition with Dabrafenib significantly suppress gastric cancer metastasis to peritoneum and liver.

## Discussion

Peritoneal metastasis is the most common and debilitating forms of metastasis in advanced gastric cancer. However, the molecular mechanism governing the occurrence of gastric cancer peritoneal metastasis remains poorly understood [21]. In this study, we demonstrated that gastric cancer peritoneal metastases have a unique gene expression profile compared to primary gastric tumors and normal stomach tissue by transcriptomic sequencing. We first identified LIMK1 as a top overexpressed gene in gastric cancer peritoneal metastasis, examined its functional role in metastatic progression and demonstrated the therapeutic value of targeting LIMK1 in inhibiting gastric cancer peritoneal metastasis.

Metastatic cascade is a multistep process whereby cancer cells detach from primary tumor, migrate and attach to distant peritoneum, followed by invasion into sub-peritoneal tissues and cell proliferation to form detectable metastasis [22, 23]. Our transcriptome analysis of paired peritoneal metastases, primary gastric tumors and normal stomach tissues revealed outlier genes selectively up-regulated in metastatic disease. Independent cohort validation showed that LIMK1 is a top gene candidate overexpressed in metastatic gastric cancer. We further proved the function of LIMK1 in promoting cell migration and invasion in gastric cancer cells (MKN74 and BGC823) in vitro; and in xenograft nude mice models of peritoneal and liver metastasis in vivo. In line with our discovery, LIMK1 has been shown to promote progression of multiple cancers including breast cancer, colorectal cancer, ovarian cancer, by inducing cell proliferation, migration or invasion [16, 18, 24, 25]. These data indicate that LIMK1 promotes gastric cancer peritoneal metastases through inducing cell migration and invasion.

Cytoskeletal remodeling is closely correlated with tumor migration, invasion and metastasis [26–28]. LIMK1 plays an essential role in regulation of actin filament dynamics by phosphorylating and inactivating the actin depolymerizing factors ADF/cofilin, leading to stabilization of the actin cytoskeleton [11, 12]. Consistent with these findings, we demonstrated that either knockout or pharmacological inhibition of LIMK1 repressed cofilin phosphorylation. Cofilin, a direct target of LIMK1, is a key regulator of actin dynamics and cell motility and invasiveness [11, 12, 29]. Our results collectively show that LIMK1-cofilin signaling underlies the promoting effect of LIMK1 on GC cell migration and invasion.

Although tumor metastasis is the major cause of cancer-related deaths, the development of druggable targets against tumor metastasis lags behind that for primary cancers. Based on our findings showing the role of LIMK1 in GC peritoneal metastases, we speculated that LIMK1 might be a therapeutic target. Dabrafenib is originally a BRAF mutant inhibitor with in vitro IC_50_ values of 0.65–1.84 nM. Dabrafenib has an IC_50_ value of 15 nM for LIMK1, and also targets additional kinases such as NEK11 and SIK1 with lower affinity (>20 nM) [17, 30]. Kinome scan showed that LIMK1 activity was reduced by 99.4% with 10uM dabrafenib [31]. Indeed, we identified that Dabrafenib could effectively inhibited cell migration and invasion in vitro. Moreover, we demonstrate that Dabrafenib IP injection attenuated peritoneal metastases and liver metastases in vivo using xenograft nude mice models. Consistent with on-target inhibition of LIMK1, Dabrafenib decreased p-cofilin levels in a dose-dependent manner, indicating that DAB reduces migration and invasion of GC cells through LIMK1-ADF/cofilin pathway. On the contrary, we found that DADS, another prototypical LIMK1 inhibitor [18, 19, 32], failed to inhibit LIMK1 and cofilin phosphorylation in GC cells. Accordingly, DADS had no inhibitory effect on cell migration and invasion. Taken together, Dabrafenib suppressed gastric cancer peritoneal metastasis through inhibition of LIMK1.

In addition to Dabrafenib and DADS, specific LIMK1 inhibitors SR7826, LIMKi3 and T56-LIMKi have also been shown to impede activity of LIMK1 on p-cofilin in prostate tissues, neurons and embryonic fibroblasts [33–36]. These inhibitors are potential drug candidates for anti-LIMK1 therapy. In line with the role of LIMK1 in GC metastasis, previous studies showed an association of LIMK1 expression and poor patient survival [36].

Encouraging progress has been made in the IP administration of chemotherapeutic agents for treatment of gastric cancer peritoneal metastasis [21, 37, 38]. There are several advantages of IP chemotherapy over traditional intravenous chemotherapy. Intraperitoneal administration of drugs (1) functions immediately on both metastatic lesions on the peritoneal surface and free tumor cells in the peritoneal cavity; (2) generates higher drug concentration in the abdominal cavity [39]; (3) prolongs half-life in the peritoneal cavity and reduces systemic toxicity [40]. We believe that Dabrafenib can be a potential drug candidate for IP chemotherapy of gastric cancer patients with peritoneal metastasis.

Although LIMK1 promotes cancer cell metastasis, it also plays an important role in the nervous system. LIMK1 is involved in spine development, axon growth and axon regeneration [41–44], and abnormal spine morphology was found in LIMK1 knockout mice [45]. Abnormal LIMK1 expression is also related to mental disorders like the Williams syndrome and Parkinson’s disease [45, 46]. Hence, the possible adverse effects of LIMK1 inhibition on the nervous system should be taken into consideration prior to its application to cancer treatment.

In summary, transcriptome profiling has unveiled LIMK1 as a novel promoter of gastric cancer peritoneal metastasis. LIMK1 mediates metastasis by promoting phosphorylation of its downstream target cofilin, leading to increased gastric cancer cell migration and invasion. Finally, we identified Dabrafenib as a LIMK1 inhibitor that can suppress gastric cancer peritoneal metastasis in experimental models.

## Materials and methods

### Patients and human samples

Two independent cohorts of gastric cancer patients were included in this study. Cohort I included 6 paired normal stomach, primary gastric cancer tumors and peritoneal metastases tissues of patients from Prince of Wales Hospital, Shatin, Hong Kong and Tianjin Medical University Affiliated Cancer Hospital. Cohort II included 29 paired primary gastric cancer tumor and peritoneal metastasis tissues of patients from Fourth affiliated Hospital of Hebei Medical University. All patients provided informed consent for obtaining the tissue specimens. This study was approved by the Clinical Research Ethics Committee of The Chinese University of Hong Kong, Tianjin Medical University Affiliated Cancer Hospital and Hebei Medical University, respectively.

### RNA isolation and RNAseq

RNA samples were isolated from frozen human tissues using TRIzol reagent (Thermo Fisher Scientific) following manufacturer’s instructions. For RNAseq, 24 samples were sequenced by using Illumina HiSeq platform (BGI, china), generating about 11.56 G bases per sample. Adapters in RNA-SEQ reads were trimmed by cutadapt (version 1.18) followed by mapping on human reference (GRCh38) by HISAT2 (version 2.1.0) with default options. Transcript assembly and transcript quantification were performed by StringTie (v1.3.5) with default options. Gene expression levels were calculated as FPKM (Fragments per Kilobase of transcript per Million mapped reads) using DESeq2.

To assess the sample quality, we performed correlation (Spearman) analysis on samples from the same group, and between our cohort (primary gastric cancer group, *n* = 9) and TCGA primary gastric cancer cohort (*n* = 19). The mapping ratio on the human genome were also taken into consideration. We have found that 1 sample (Patient 2-PT) had the poor quality, which had low correlation with other samples and had poor mapping ratio on human genome. Finally, 23 samples have passed the sample quality and are used for differential analysis by DESeq2.

### Immunohistochemistry (IHC)

For IHC analysis, following primary antibodies were used: Anti-S100A13 (1:500, Abcam), Anti-BCHE (1:500, Abcam), Anti-LIMK1 (1:200, Abcam), Anti-ITGA4 (1:200, Abcam). Tissue specimens were deparaffinized, rehydrated, and processed for antigen retrieval in boiling citrate buffer for 6 min. Slides were blocked in blocking reagent (Millipore) for 10 min, incubated with primary antibodies in 4 °C overnight, washed with Tris-buffered saline with 0.1% Tween 20 (TBS-T), then incubated with secondary antibody (Millipore) for 45 min. HRP/DAB kit (Millipore) was used to visualize stanning. Images of IHC were acquired by microscope using 10x and 20x objectives. IHC staining was scored by percentage positive area and intensity as follows: 0, no staining; 1, <10% positive, moderate or strong intensity; 2, 10–50% positive, moderate or strong intensity; 3, >50% positive, moderate intensity; and 4, >50% positive, strong intensity.

### Gastric cancer cell lines and cell culture

MKN74 cells were purchased from the Korean Cell Line Bank (Seoul, Korea). BGC823 cells were acquired from the Cell Bank of Chinese Academy of Sciences (Shanghai, China). All cells were routinely cultured in DEME (Gibco BRL, Rockville, MA) supplemented with 10% fetal bovine serum and 1% Anti-Anti. Stable LIMK1 knockout gastric cancer cell lines were established using lentivirus infection and selection with 2 ng/ul puromycin.

### CRISPR/Cas9 knockout

To generate CRISPR/Cas9 plasmids targeting LIMK1, 2 oligos of single-guide RNAs were synthesized at BGI Group (Beijing, China). Lentiviral transfer plasmids were constructed following the protocol described by Shalem et al. [47], then transfected into 293 T cells together with two packaging plasmids psPAX2 and pMD2.G to package lentivirus. sgRNA oligo sequences were: sgRNA 1, 5′-TGACGGGGACACCTACACGC-3′; sgRNA 2, 5′-CGCTATGGCGAGTCCTGCCA-3′.

### LIMK1 knockdown by siRNA

siRNAs were synthesized by GenePharma (Shanghai, China). The sequences were: siRNA 1, sense 5′-GGAUGGCACCUGAGAUGAUTT-3′, antisense 5′-AUCAUCUCAGGUGCCAUCCTT-3′. siRNA 2, sense 5′-CCAUGGACUUUGGCCUCAATT-3′, antisense 5′-UUGAGGCCAAAGUCCAUGGTT-3′. Transfection of siRNAs was performed with Lipofectamine 2000 following the manufacturer’s protocol.

### Wound healing cell migration assay

Cells were cultured as a confluent monolayer in six-well plates, and carefully scratched with a 10uL pipette tip. After washing with PBS to remove detached cells, images in 12 distinct wound fields were captured at respective time points (0, 24, 48 and 72 h) by microscope using 5x objectives.

### Transwell assay (cell migration and invasion assay)

Chambers without or with matrigel (Corning, NY, USA) were used to investigate migration or invasion ability of cell, respectively. To investigate cell migration ability, 2 × 10^5^ cells suspended in 100 mL serum-free medium were seeded onto to the upper chambers without matrigel, put in 24-well plate containing 600 mL of medium with 10% FBS in the lower chambers. Cells were then incubated in the 37 °C incubator. At the end point of experiment, cells on the upper side of the chambers were removed thoroughly with cotton swab, and cells on the bottom side of the membrane were stained with 0.1% crystal violet. As for cell invasion assay, chambers were pre-incubated according to the manufacturer’s recommendations before seeding the cells, then performed in the same manner as cell migration assay. All the experiments above were performed in duplicates. Cell numbers of 5 randomly selected fields were counted under the microscope. Results represent the average number of cells in each field.

### Western blot

For western blot, the following primary antibodies were employed: Anti-LIMK1 (1:1000, Abcam), Anti-cofilin (1:1000, Cell Signaling), Anti-p-cofilin (1:1000, Cell Signaling), Anti-GAPDH (1:2000, Santa Cruz). Proteins were prepared by using CytoBuster Protein Extraction Reagent (Millipore, MA, USA). After separation by SDS-polyacrylamide gel electrophoresis, proteins were transferred to PVDF membrane, incubated with primary antibodies at 4 °C overnight and secondary antibodies. Proteins were visualized using Clarity Western ECL Substrate (Bio-Rad, USA).

### Animal models of gastric cancer metastasis

Four-to-six weeks old male nude mice were used for gastric cancer peritoneal metastasis model. The number of animals were determined based on our experience. The investigators were blinded to group allocation during the experimental procedures. MKN74 cells (1 × 10^7^) with stably LIMK1 knockout or negative control were injected into the abdominal cavity of the nude mice (*n* = 5 per group). Mice were then given IP injection of dabrafenib (DAB, 5 mg/kg) or DMSO blank control every 3 days started from day 3. Twenty-eight days after injection, mice were sacrificed and examined. The peritoneal metastases were counted in a blinded manner, dissected and paraffin embedded. Sections were stained with hematoxylin and eosin (H&E).

Four-to-six weeks old weeks female nude mice were used for gastric cancer liver metastasis model. MKN74 cells (1 × 10^7^) with stable LIMK1 knockout or negative control were injected into the spleens of the nude mice (*n* = 5 per group). The spleens were resected after injection. Mice were then given IP injection of dabrafenib (DAB, 5 mg/kg) or DMSO blank control every 3 days started from day 3. Twenty-eight days after injection, the mice were sacrificed and examined. The liver metastases were counted in a blinded manner, dissected and paraffin embedded. Sections were stained with H&E. All animal experimental procedures were approved by the Animal Ethics Committee of the Chinese University of Hong Kong.

### Statistical analysis

All results were presented as mean ± SD. All in vitro experiments were performed in triplicates and were repeated at least twice independently. Statistical analysis was performed by GraphPad Prism 8.0 (GraphPad, La Jolla, CA, USA). Student’s *t* test was performed to compare the means between two groups. Wilcoxon signed-rank test was performed to compare IHC score between peritoneal metastases and primary tumor tissue. Two-way ANOVA test was performed to compare the body weight change of mice. Variance between the groups were statistically compared. P values < 0.05 were considered to indicate significance. *P < 0.05; **P < 0.01; ***P < 0.001; ****P < 0.0001.

## Supplementary information

Supplemental figure legends

Figure S1

Figure S2
