# Supplementary material for: LIMK1 promotes peritoneal metastasis of gastric cancer and is a therapeutic target
Source: Oncogene. 2021 Apr 21;40(19):3422–33. doi: 10.1038/s41388-021-01656-1 (PMC8116207; doi:10.1038/s41388-021-01656-1)
Supplement: Supplementary file 1 — Supplemental figure legends [file 41388_2021_1656_MOESM1_ESM.docx]

**Supplementary figure 1.** IHC staining of samples from validation cohort showed the expression of both BCHE and S100A13 have no significant difference between peritoneal metastases (PM) compared to primary tumors (PT).

**Supplementary figure 2.** LIMK1 expression in TCGA GC cohort. **(A)** LIMK1 mRNA expression is higher in GC patients with copy number gain. **(B)** LIMK1 mRNA positively correlates with its DNA copy number (P<0.0001). **(C)** LIMK1 mRNA expression is independent of BRAF status.
