# Supplementary figures and images for: LIMK1 promotes peritoneal metastasis of gastric cancer and is a therapeutic target
Source: Oncogene. 2021 Apr 21;40(19):3422–33. doi: 10.1038/s41388-021-01656-1 (PMC8116207; doi:10.1038/s41388-021-01656-1)

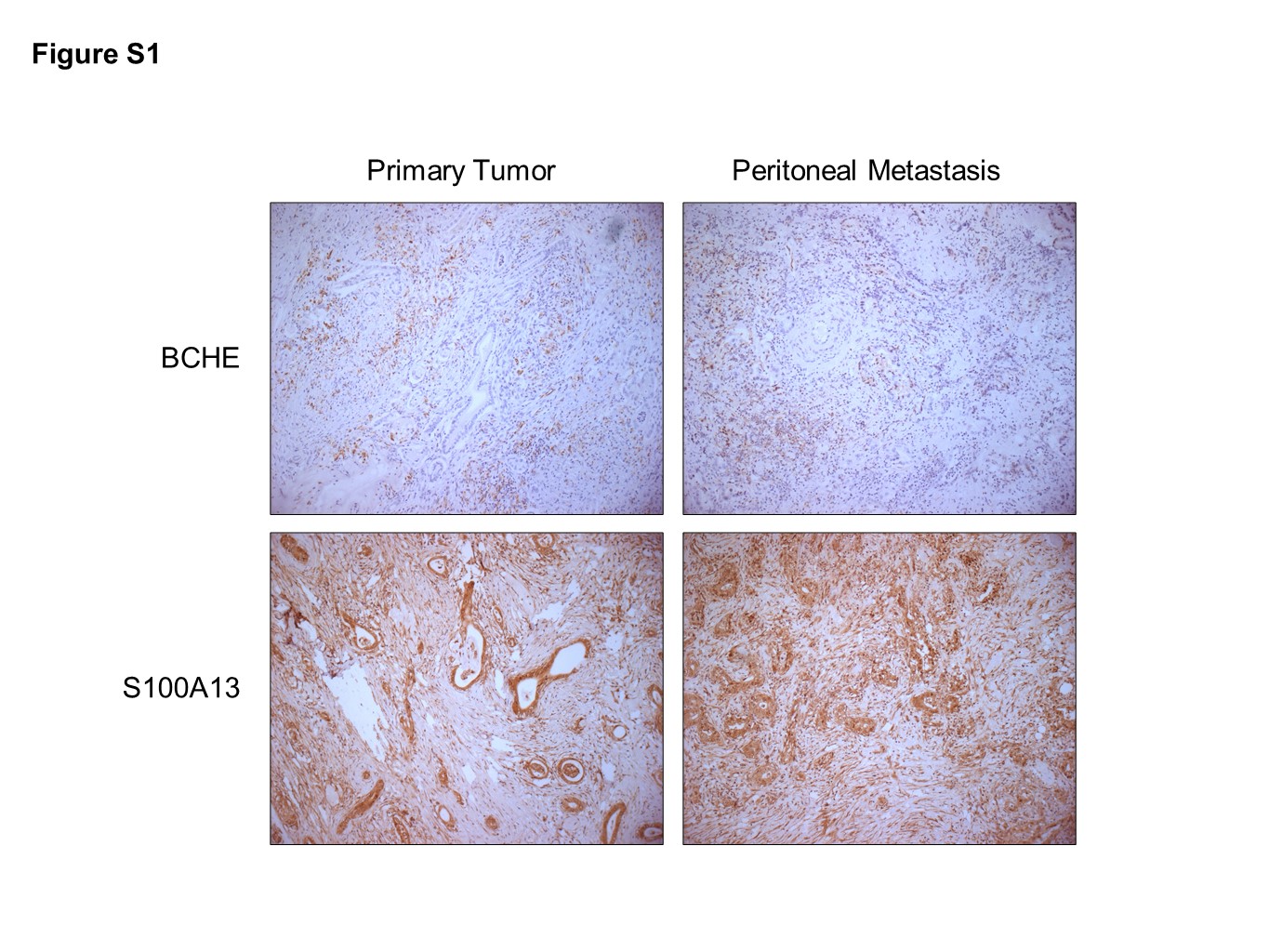

Supplement: Supplementary file 2 — Figure S1 [file 41388_2021_1656_MOESM2_ESM.jpg]

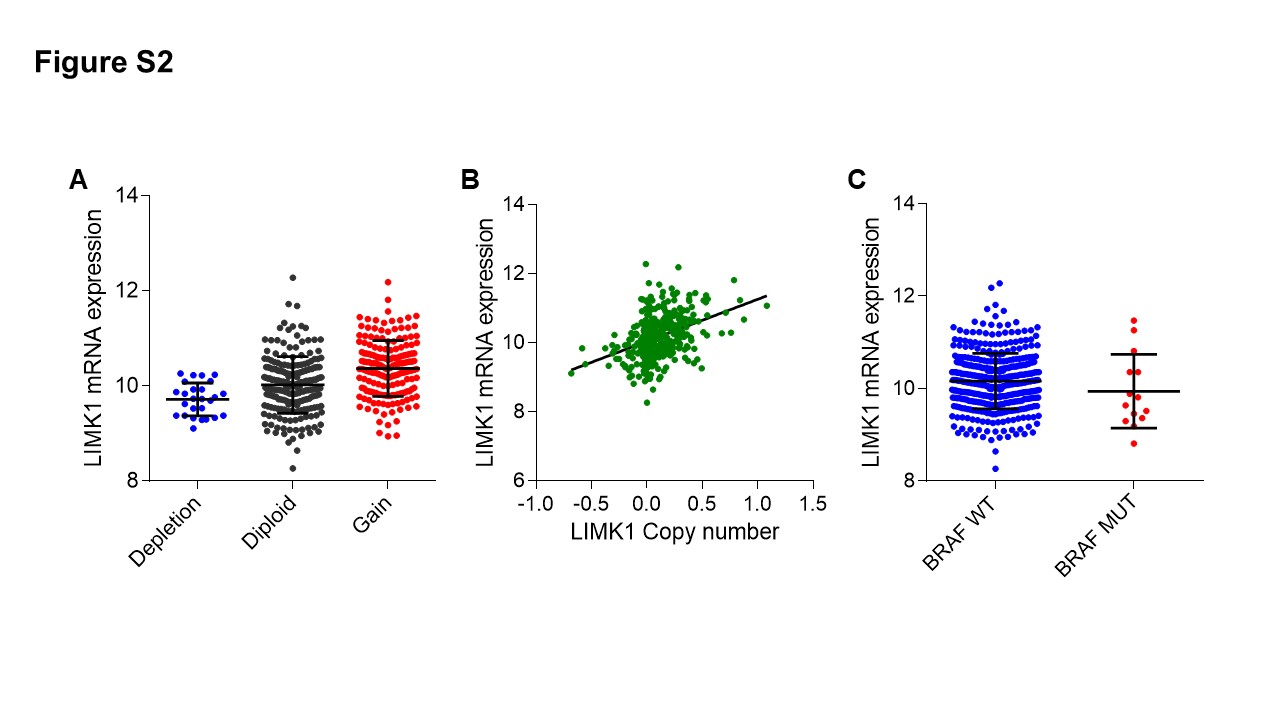

Supplement: Supplementary file 3 — Figure S2 [file 41388_2021_1656_MOESM3_ESM.jpg]
